# FEDERAL AND STATE POLICIES AND STAKEHOLDER VIEWS ON MEDICAID NURSING HOME PAYMENT

**DOI:** 10.1093/geroni/igae098.2002

**Published:** 2024-12-31

**Authors:** Edward Miller, John Bowblis, Elizabeth Simpson, Sara Karon, Iara Oliveira, Judith Dey, Marie Squillace, Marc Cohen

**Affiliations:** University of Massachusetts Boston, Boston, Massachusetts, United States; Miami University, Oxford, Ohio, United States; University of Massachusetts Boston, Boston, Massachusetts, United States; RTI International, Research Triangle Park, North Carolina, United States; Office of Behavioral Health, Disability, and Aging Policy (BHDAP), Washington, District of Columbia, United States; US Department of Health and Human Services, Washington, District of Columbia, United States; Office of the Assistant Secretary for Planning and Evaluation, Washington, District of Columbia, United States; University of Massachusetts Boston, Boston, Massachusetts, United States

## Abstract

How much nursing homes (NHs) are paid by Medicaid is determined at the state level. Medicaid coverage of NH care is a significant expenditure for states. Consequently, states design Medicaid payment models to achieve desired policy objectives related to NH costs and quality, access to care, payment equity, service capacity, and budgetary control. Setting Medicaid payment rates too high can lead to excess profits at the expense of taxpayers with unknown implications to improvements in quality and staffing levels. At the same time, NHs need financial resources to pay nursing staff and invest in other quality improvement efforts to assure proper care. The industry claims that NH costs are not fully covered by their Medicaid payments. Consumer advocates argue that NHs are already adequately paid, and profit incentives potentially divert funds away from resident care. There is no federal requirement that Medicaid payments cover the full cost of care.

